# New Realization of Disaster Risk Reduction Education in the Context of a Global Pandemic: Lessons from Japan

**DOI:** 10.1007/s13753-021-00337-7

**Published:** 2021-03-25

**Authors:** Rajib Shaw, Aiko Sakurai, Yukihiko Oikawa

**Affiliations:** 1 Graduate School of Media and Governance, Keio University, Fujisawa, 252-0882 Japan; 2 Department of Social Sciences, Toyo Eiwa University, Yokohama, 226-0015 Japan; 3 International Research Institute of Disaster Science, Tohoku University, Sendai, 980-0845 Japan; 4 Center for Ocean Literacy and Education, University of Tokyo, Tokyo, 113-0032 Japan

**Keywords:** Citizen behavior, Disaster risk reduction education, Education for sustainable development, Global COVID-19 pandemic, Japan, School-community-family linkage

## Abstract

The global COVID-19 pandemic has challenged different development sectors, including education. In this article, two main analyses are provided: one on the biological hazards of the pandemic in the context of the Sendai Framework for Disaster Risk Reduction 2015−2030, which analyzes the overall impacts on the education sector. Then we discuss the overall impact on education sectors, with specific focus on disaster risk reduction (DRR) education and education for sustainable development (ESD). Disaster risk reduction education and ESD are analyzed from the perspective of school-community-family linkages. Specific case analysis of COVID-19 response in the education sector is presented from Omuta City, Japan, which is considered as a champion city for ESD. Four phases of response in Omuta City are characterized with three specific foci: (1) mitigating covid impacts on educational program and participants; (2) preventing exacerbation of covid transmission within and outside schools; and (3) maintaining educational program integrity despite covid. Key lessons are summarized in the concluding section, which explore the importance of (1) educational governance (on critical decision making) during the pandemic as well as with cascading risks; (2) enhancement of school-community-family linkages as pandemic response commonalities between ESD and DRR education; (3) risk communication and citizen behavior; and (4) use of technology. We argue that integration of health and DRR education is important, that resilience needs to be redefined in terms of sustainable development goals (SDGs), and that education plays a vital role in achieving these ends.

## Introduction

Disaster risk reduction (DRR) has evolved over time through the painful experiences of different disasters, both in Japan and abroad. Living with a diverse society and coexistence with nature has been part of human history. In our ever-changing lifestyles, we have termed the co-existence as education for sustainable development (ESD) or environment education. Similarly, living with disasters and living with risk have always been part of human history, and experiential learning has been the core to educational process. We have renamed this experiential process as disaster risk reduction education, henceforth called DRR education. As many authors have argued (Shiwaku and Shaw 2008; Shaw, Takeuchi, et al. 2011; Shaw, Shiwaku, et al. 2011; Thi et al. 2012; Shiwaku et al. 2016), DRR education links school, community, and home. The ultimate goal of DRR education is to internalize risk perception and enhance preventive/preparedness actions. This has always been an evolving process. Shaw, Takeuchi, et al. (2009) proposed the KIDA (Knowledge Interest Desire Action) model as the example of process-based DRR education.

School safety has been a strong pillar of DRR education, which includes a physical part, management components, and education components (ASEAN 2016; UNISDR and Global Alliance for Disaster Risk Reduction and Resilience in the Education Sector 2017). Japan has been in the forefront of DRR education, be it in the formal sectors like schools or informal sectors such as communities. After the 2011 Great East Japan Earthquake and Tsunami, the Japanese government has started focusing on DRR education related to the importance of life. It is often said that DRR education is a lifelong education and is focused on behavioral changes (UNICEF and UNESCO 2009; Shaw and Oikawa 2014). In DRR education, the traditional focus has been on “how to evacuate” as exemplified in emergency drills. But several disasters have taught the authors that it is not just sufficient to have an education on “how to.” Rather, we need to focus more on “what to.” In that more proactive space, risk information, risk understanding, and risk perception have a critical role to play. We need to think of a more holistic perspective within which to encourage resilience in the human, natural, and socioeconomic/governance systems.

The COVID-19 pandemic is the worst biological hazard-induced disaster observed in recent memory. Its unprecedented speed and spread have affected most parts of the world. The year 2020, which was supposed to be an important milestone year for the Sustainable Development Goals (SDGs) (UN 2015), the Sendai Framework for Disaster Risk Reduction 2015−2030, and the Paris Climate Agreement, is now under the shadow of the pandemic. The pandemic has not only impacted economies at every level, but it has also hindered the achievement of the SDGs. Moreover, the cumulative effect of COVID-19 has strongly impacted national and local development. The education sector is no exception to that adverse result. In the formal education sector, all levels, including primary, secondary, and tertiary (higher education), are affected. COVID-19 has also negatively influenced nonformal and informal education through lock downs or emergency regulation announcements in most countries, and thereby prohibited direct contact between people.

The United Nations, in a recent estimate, has pointed out that the pandemic has potentially affected 1.6 billion learners worldwide, impacting 94% of the student population (UN 2020). The impact is more prominent in poor and vulnerable communities, although several developed countries have also felt the disruption with a certain amount of increased dropout rate in the schools. Educational disruptions have impacted the mid-day, in school meal, which is often considered to be the only nutritious food for school-age children in poor neighborhoods. The prolonged pandemic has also increased violence against children, especially girls. Financing education seems to be a challenge in many countries, and gaps with pre-COVID-19 education funding are increasing with a staggering estimated figure of USD 148 billion globally as per the United Nations (UN 2020) report. This report also advocated establishing resilient education systems that can be linked to sustainable development of communities and nations.

The past few months of pandemic experiences have not only changed the course of education as a whole, they have also impacted the meaning and realization of DRR education. Cascading disasters have been common in many parts of the world. We have seen several cyclonic storms hit coastal communities in which the ongoing pandemic has posed a key challenge to the evacuation process. Strong heat waves and flooding during the pandemic have posed new health-related challenges. In many countries, education related to the pandemic is considered as a “health education.” The pandemic impact goes beyond health, however, and is strongly related to the Sendai Framework for Disaster Risk Reduction 2015−2030 (Djlante et al. 2020; Shaw, Kim et al. 2020).

Within this context, this article analyzes the biological hazards perspectives of the Sendai Framework and draws a few key lessons. An analysis of DRR education in the new risk landscape follows, as well as analysis of ESD, DRR education, and the link to SDGs. Key issues of DRR education and COVID-19 impacts in Japan are also described. A specific case from Japan illustrates the need for anew realization of DRR education, linked to education for sustainable development. Finally, the article proposes key learning for DRR education in the “new normal” condition by analyzing the case of Omuta City, Japan. In this article, we argue that the new perspective of integrated DRR education can implement the lessons learned from the pandemic as well as enhance reduction of systemic, cascading risks.

## Biological Hazards and Impacts on Education Sectors

The Sendai Framework reinforces the scope of disaster risk management by expanding beyond natural hazards to include biological hazards such as epidemic and pandemic diseases. The Sendai Framework also places strong emphasis on the need to build resilient health systems through the integration of disaster risk management into the provision of healthcare at all levels and, in particular, “to enhance cooperation between health authorities and other relevant stakeholders to strengthen country capacity for disaster risk management for health” (UNISDR 2015, p. 19). The purpose of this section is to highlight that a pandemic response is not just a health response, but rather is an integrated development sector’s response to a disaster, which is caused by biological hazards.

United Nations Office for Disaster Risk Reduction (UNDRR) terminology defines biological hazards as follow (UNDRR 2017a, 2017b):

Biological hazards are of organic origin or conveyed by biological vectors, including pathogenic microorganisms, toxins and bioactive substances. Examples are bacteria, viruses or parasites, as well as venomous wildlife and insects, poisonous plants and mosquitoes carrying disease-causing agents.

**Fig. 1:**
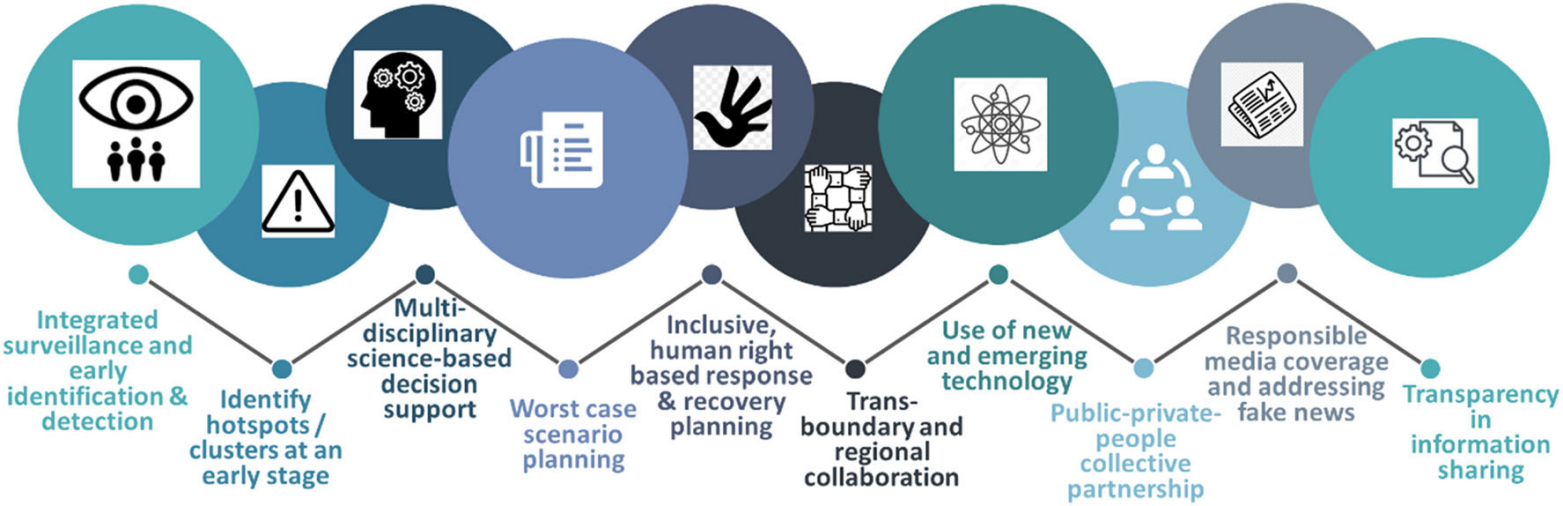
Basic steps and learning for biological hazard integration into disaster risk reduction (DRR). *Source* Shaw, Chatterjee, et al. (2020a)

In a recent study, Shaw, Chatterjee, et al. (2020a, 2020b) have identified 10 basic principles that need to be incorporated into future risk preparedness (Fig. 1). These principles define the key aspects of biological hazards: (1) risk assessment (integrated surveillance and early detection, identification of hotspots/clusters); (2) risk planning (multi-disciplinary science-based support, worst case scenario planning); (3) recovery planning (with regional collaboration); and (4) use of technologies and stakeholder participation in order to address fake news and infodemics (information as the key of epidemic, mentioned by WHO Director General in January 2020) by means of information sharing, which is an important component of risk communication. Risk communication is also an essential element of disaster risk management (DRM) because it shapes people’s perceptions of risk and influences their actions with respect to disaster preparedness and disaster response. Priority Four of the Sendai Framework (UNISDR 2015) specifies the importance of investing in disaster risk communication along with multi-hazard forecasting and early warning systems, developing these systems with participatory process, tailoring them to the needs of users, and broadening release channels for disaster early warning information. These principles can be adopted for the disaster risk reduction process, and the education sector can also learn and adopt some of these goals and practices.

The education sector is differentially affected by COVID-19. The sector needs to provide a unified response with: (1) mitigating disease impacts on students, staff, and support personnel within its direct purview; (2) adopting practices that do not exacerbate disease transmission in the local community; and (3) maintaining its educational mission in creative and innovative ways, despite being seriously impacted, based on its unique knowledge resources. The education sector can help reduce local impacts by generating health education that increases student and community awareness of disease ecology and preventive public health practices. In this way the education sector plays a critical role in risk communication about outbreaks, epidemics, and pandemics.

Education also supports development of rich human resources by imparting required training and skills. Higher education institutions (HEIs) are resourceful in conducting research on various aspects linked to an outbreak and in increasing risk awareness. During the COVID-19 outbreak, the HEIs can play an important role in response to the disease outbreak, spread, and status. This has been highlighted by examples such as the role of Johns Hopkins University in developing an online dashboard that documents the spread of the infection or the role played by students at the Indian Institute of Technology, Roorkee in developing low-cost ventilators (Izumi et al. 2020).

The education sector is one of the most frequently impacted service sectors. The main reason is the closure of schools and absenteeism of teachers. The propensity of children to become infected with particular virus infections has led to the closure of schools in the past. This disturbs the academic calendar and adds stress to parents and students alike. The education sector has embraced online tools, redefining how learning takes place by opening up the traditional classroom. The effectiveness of online education in student learning and its impact on care providers’ regular responsibilities (for example, work duties and care responsibilities) should not be neglected. Issues of undernutrition in low income groups in developing countries and obesity globally not only put the children at high risk of infection but also may expose them to long-term developmental, psychological, physical, and emotional complications (Dunn et al. 2020). A pandemic may expose children to higher risks of abuse, neglect, and other issues around child protection (UNICEF 2020). A biological hazard also may often be an opportunity for higher education institutions and research organizations to engage in developing new tools, encourage research and innovation, and find new funding opportunities.

## Disaster Education in the New Risk Landscape

COVID-19 has brought new challenges as well as opportunities in the education sector, similar to other sectors like health, livelihoods, and so on. In the new risk landscape, where the biological hazards continue over a period of time, and there are risks of cascading hazards, education sector’s role becomes more important for formal, informal, and nonformal education.

### Implication of COVID-19 Pandemic to Education

Early resumption and continuity of educational services after a disaster is listed as one of the global targets in the Sendai Framework for Disaster Risk Reduction 2015−2030. The COVID-19 pandemic gave enormous global shocks to children’s education. Different from a natural hazard and disaster, the pandemic affects children’s education all over the world almost simultaneously. The impact of educational services disruption by the COVID-19 pandemic extends from lost learning opportunities to children’s learning outcomes, increasing dropouts, and disruption of school nutrition programs. Isolation of children from schools could also affect their mental health. In addition, economic shock puts pressures on households that could decrease educational expenses. Fiscal pressure may lead to reduction in education investments, impacting learning environments and quality of education (World Bank 2020; UN 2020).

With the COVID-19 virus pandemic, each country has taken all possible actions to prevent disease spread, and international cooperation has been explored to overcome the crisis. Lockdown measures were utilized in cities in many countries. In mid-June of 2020, lockdowns started to be lifted gradually in some countries although the spread of the coronavirus and emergence of new varieties continue. New lockdowns were imposed in many countries, which were undergoing a third wave between November and December 2020. In Japan, schools were faced with sudden closures in the beginning of March and then again in June 2020, just as the country was at the phase of safe school resumption. Although people tried to return to their normal daily life, it was not possible to resume the same life as before the pandemic. To turn the COVID-19 pandemic crisis into opportunities in the education sector, the international community started to discuss how educational systems could be built back stronger and more equitable than before (UN 2020; World Bank 2020). Since disaster education is one of learning activities, it is worthwhile examining what disaster risk reduction education could learn from the COVID-19 pandemic in the new risk landscape.

### Living with COVID-19 Risk

Risk communication has been playing a vital role in management of the COVID-19 pandemic. Governments and public health agencies in each country have been informing the public about what the COVID-19 virus is, what the symptoms of the virus are, how to avoid catching the virus, what measures to take when the suspicion of infection arises, and so on. Public health education also involves multiple messages, not strictly about risk, that express concerns, opinions, or reactions to risk messages or to legal and institutional arrangements for risk management (Natural Research Council 1989). The goals of risk communication in public health are to share information vital for saving life, protecting health, and minimizing harm to self and others throughout a society. Regarding the COVID-19 virus, each individual needs to change their behavior to avoid personal risk of infection, but also the whole society needs to take actions against societal risk to prevent disease spread in the society. To respond to a societal risk requires all citizens, each of whom has a different level of risk perception, to participate in discussions to reach a social agreement on appropriate response to a societal risk. Risk communication is recognized as multidirectional communication and engagement with affected populations so that they can take informed decisions to protect themselves and their loved ones. This can and should utilize the most appropriate and trusted of channels of communication and engagement (WHO 2014). It could be said that infectious risk of COVID-19 is a new risk, which people perceive as “dread” and “unknown.” The higher the dread factor levels and the higher the perceived unknown risks, the more people want to see such current risks reduced, and the more they want to see strict regulation employed to achieve the desired reduction in risk (Slovic 1987).

## Education for Sustainable Development, Disaster Risk Reduction Education, and Sustainable Development Goals

ESD, DRR education, and SDGs are inter-connected, and often considered as the part of the same coin. Oikawa (2016) has demonstrated the linkages among the three types of education, and the connectivity goes to the school community linkages. This section provides a brief overview of these three types of education process.

### Improving Disaster Risk Reduction Education through Education for Sustainable Development

Faced with the global COVID-19 infection, social systems and lifestyles need to adjust to extreme change. Disaster risk reduction education should also change its concept and method towards achieving a sustainable society. Education for sustainable development brings new perspectives to DRR education. As a lesson learnt from the 2011 Great East Japan Earthquake and Tsunami and ESD practice in Kesennuma City, Miyagi Prefecture, Japan, ESD brought new innovations to DRR education from the perspective of the sustainable development concept. This achievement was attained by improving the quality of DRR education, fostering DRR ability and attitude, and building networks and partnerships for DRR (Oikawa 2014c). In the context of DRR and ESD values, such concepts as respect for lives, human security, life together (coexistence), and building a sustainable society (Build Back Better) emerged. Education for sustainable development also transforms learning styles of DRR education. Education for sustainable development recommends learning that is inquiry-based, problem-solving, experience-based, project-oriented, community-based, and integrated. Adoption of those ESD learning methods into DRR education should further improve the quality of DRR education. As a result, through the improvement based on ESD, DRR education can foster DRR abilities and attitudes that become the building blocks of disaster management (Oikawa 2014b).

### Education Achieving the Sustainable Development Goals

**Fig. 2:**
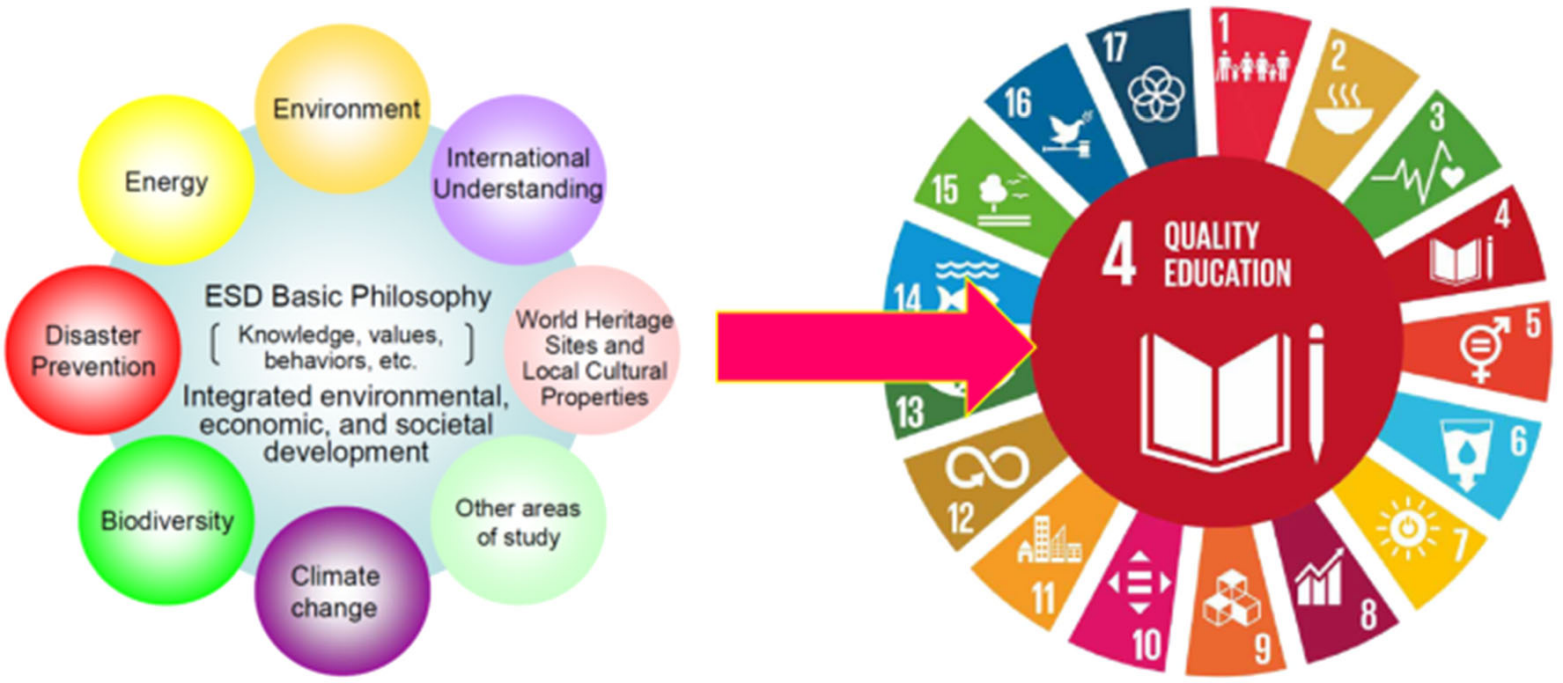
Education for sustainable development (ESD) towards achieving sustainable development goals (SDGs). *Source* Left: Japanese National Commission for UNESCO (https://www.unesco.emb-japan.go.jp/htm/jpcommissionunesco.htm); right: UNESCO (https://en.unesco.org/sustainabledevelopmentgoals)

Sustainable development goals ensure inclusive and equitable quality education and promote lifelong learning opportunities for all at Goal 4. Education is at the heart of the 2030 Agenda for Sustainable Development and is essential for the success of all SDGs (Fig. 2). Especially, ESD is a key enabler of all the other SDGs, so that the overall objective of ESD for 2030 is to build a more just and sustainable world through the achievement of the 17 SDGs. Pursuant to the achievement of SDGs, the United Nations Educational, Scientific and Cultural Organization (UNESCO) submitted a proposal for a framework, entitled Education for Sustainable Development: Towards Achieving the SDGs (ESD for 2030),^1^ as a 10-year follow-up to the Global Action Programme on Education for Sustainable Development (2015−2019),^2^ and this proposal was adopted at the UN General Assembly in December 2019. The ESD for 2030 therefore proposes to strengthen ESD’s contribution to all SDGs, with particular focus on helping attain Goal 4. The UNESCO document also stresses that future education—the Education 2030 agenda—should place emphasis on the contribution of learning content to the survival and prosperity of humanity (UNESCO 2019). In this context, the COVID-19 pandemic is an unprecedented disaster that threatens human existence, and a common issue to be solved by all humans. Therefore, the response to prevent the COVID-19 pandemic should be implemented in the context of SDGs, and as result, its process leads to the achievement of SDGs. To realize this, education, especially DRR education based on ESD, should make a great contribution through capacity development.

### Overview of Disaster Risk Reduction Education and Sustainable Development Goals

Sustainable development goals mention DRR at Goal 1, 11, and 13. In the SDGs context, it is possible to promote DRR education targeting DRR-related goals above, mainly Goal 11, and combining with SDG 4 focused on education. Yet DRR education should not be limited to SDGs 4 and 11. Disasters have serious impacts on other issues such as poverty (Goal 1), food and hunger (Goal 2), clean water and sanitation (Goal 6), energy (Goal 7), infrastructure (Goal 9), and so on. Many disasters, such as storm, flood, drought, tsunami, and landslide, occur from climate change (Goal 13), rising seawater temperature and tsunami (Goal 14), and deforestation and desertification (Goal 15). Therefore, promoting DRR education centered on DRR (Goal 11) and education (Goal 4) in relation to other goals can contribute to achieving all SDGs. The COVID-19 pandemic is also related to many other SDG goals, such as poverty, food, health, sanitation, economic growth, among others. Thus responses to prevention of infection are congruous with the aims of DRR education. This perspective is crucial for education of COVID-19 pandemic.

## Japan’s Disaster Risk Reduction Education and COVID-19 Response

Disaster education is considered one of the components of safety education in Japan, which aims to promote a person’s ability to overcome normalization bias that inhibits realistic disaster threat risk assessment, prediction, and avoidance, and thus makes it possible to take pragmatic, proactive action against a looming threat (MEXT 2019). Education for risk communication is scattered among environmental education, safety education, information education, and consumer education in the current national curricular guidelines in Japan. But the topic of natural hazards and disasters is still dealt with in the confines of individual subject areas. That is, the natural environment and the social environment are covered separately in science education and social studies education, respectively. Because of the different goals and characteristics of these two subject areas, natural hazards and disasters have not been comprehensively covered despite the need for a cross-curricular approach (Fujioka 2016).

To overcome the division in the curriculum, ESD is one of the solutions because integrated efforts in numerous connected fields are required to achieve the construction of a sustainable society. Experiential learning is also a useful approach for children to learn about a concept of risk and risk management that is difficult even for the adults to understand. Since the scale and degree of a disaster could be different according to the natural and social vulnerabilities of each affected area, a place-based experiential learning could be an effective approach. Disaster education is not only limited to formal education. Extracurricular and continuous education activities in communities could help the participants, regardless of their age, to build trustful relationships among themselves, and to understand their own community’s society and nature holistically. These educational efforts could energize participatory disaster risk management by any local community trying to find, through discussion, solutions that reduce disaster risk.

From the experience of the 2011 Great East Japan Earthquake and Tsunami disaster, participatory disaster risk management by the local community has been proven to be an effective way of communicating risk. When a local community was involved in planning for disaster preparedness, and people took ownership of their own safety plans, they were better prepared and better able to take the necessary actions to protect themselves. Successful risk communication occurs when there is holistic learning, facilitation, and trust. In contrast, risk communication in the Fukushima nuclear accident was regarded as a case of failure in terms of the timing of information released, the accuracy and details of the information provided, and the trust and credibility of the information sources cited (Shaw 2012).

Having only basic or minimum understanding of risk, how did people respond to the COVID-19 risk in the case of Japan? Risk perception is different in each country based on the society’s culture and the government’s actions. During a state of emergency period, the Japanese people obeyed the stay-home request without any law enforcement (Shaw, Kim et al. 2020). Commuting by jam-packed trains is a notorious daily-life scene in Tokyo, but empty seats were found in commuter trains after repeated requests from the government to reduce the number of people in public place by 80% compared to before the state of emergency declaration. People kept social distances, wore masks in public spaces, and washed hands and gargled when they returned home (Tashiro and Shaw 2020). Not only did individual behavior change to avoid infectious risk, people also refrained from becoming spreaders of the coronavirus. In this short period, the Japanese people and society experienced first-hand the behavioral changes needed to avoid COVID-19 risk. But discrimination and stigma against hospital workers and hoarding certain products also occurred in the society; these behaviors were assumed to be caused by fear of contamination and rumors about potential shortages potentially caused by the unknown and the invisible coronavirus.

## Case of Omuta City

Omuta City in Fukuoka Prefecture has taken deep interest and leadership in promoting ESD through SDG activities. Working closely with the schools, local communities, academics, and nongovernment organizations, as well as business sector, the city has established a collaboration scheme for DRR, ESD, and SDG.

### Building the Linkage of School and Community through Education for Sustainable Development and Sustainable Development Goals

**Fig. 3:**
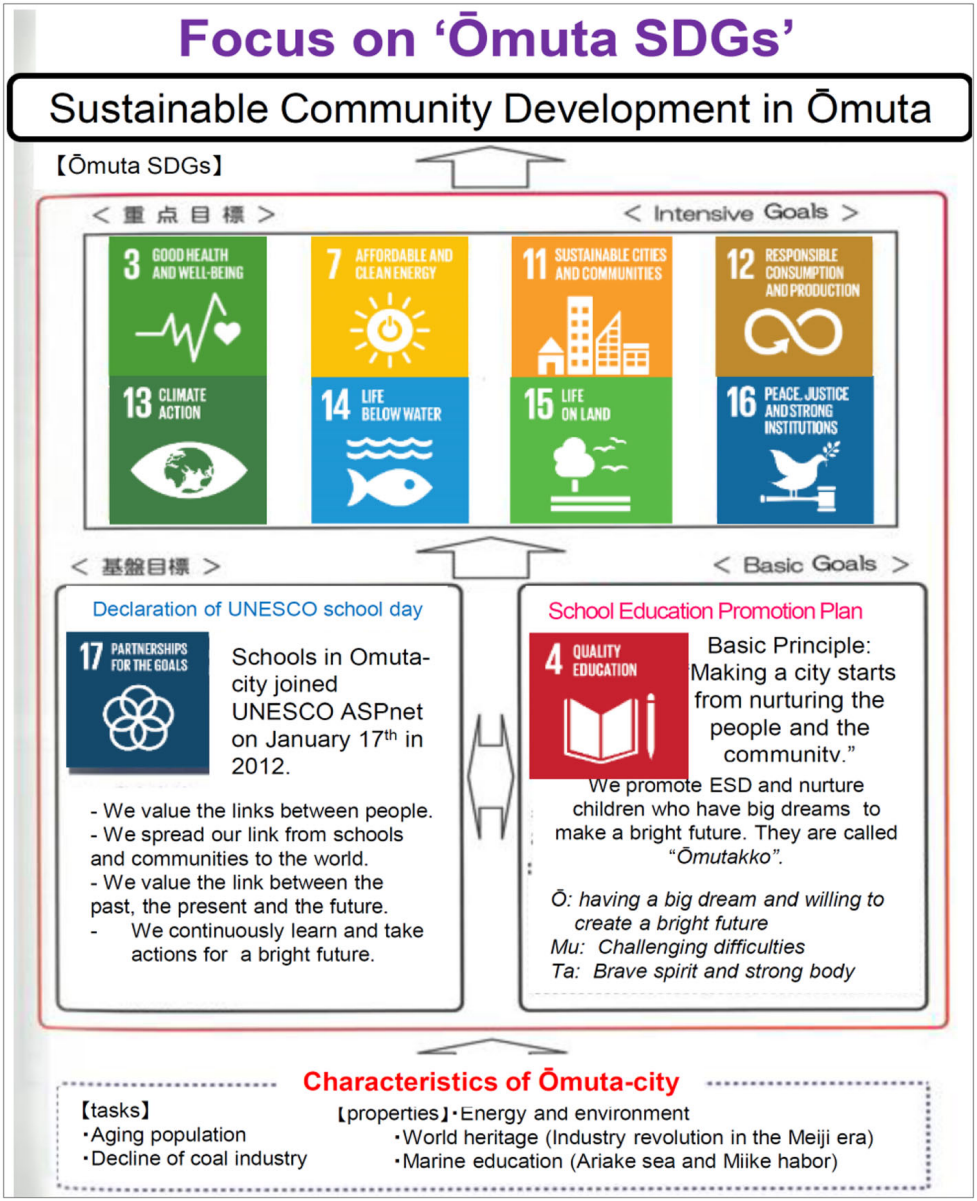
Omuta version sustainable development goals (SDGs). *Source* Omuta City (https://omutacityzoo.org/sdgs)

The Omuta City case is analyzed as a good example of school and community linkage practiced through ESD. Omuta City is located in the southeast of Fukuoka Prefecture, adjacent to the Ariake Sea and close to the Kumamoto Prefecture boundary. Omuta’s economy flourished along with the coal industry as long as coal was Japan’s primary fuel source. But in 1997, faced with competition from alternative fuel sources, the Miike Coal Mines closed, Omuta’s industry declined, and population fell by nearly 50% as the city’s work force sought jobs elsewhere. Today, utilizing the coal mine-related resources, which are World Heritage sites, Omuta is advancing its own Omuta-brand SDGs to enhance education that promotes sustainable communities. With the slogan “Omuta, an UNESCO Associated School Town,” all of the city’s 30 elementary schools, junior high schools, and special needs schools became UNESCO Associated Schools (ASPnet School) in 2011 and has been practicing ESD. The Omuta City Board of Education (BOE) has built an ESD Consortium comprising local companies, nongovernment/nonprofit organizations, the University of Teacher Education Fukuoka, and others. Through building the ESD Consortium, the linkage of school and community in Omuta City has been strengthened (Oikawa 2016). In 2018, the city developed “Omuta version SDGs” by adapting UN SDGs to align with local issues. The consortium selected several sustainable development goals and clarified those children’s competencies to be nurtured to achieve the goals based on former ESD practices (Fig. 3). Schools in the city also promote DRR education utilizing their linkage with local communities (Omuta BOE 2018).

### Educational Response to COVID-19 in Omuta City

The Omuta City Board of Education (BOE) has been conducting responses to COVID-19 infection by collaborating with schools, parents, and local communities since the COVID-19 pandemic occurred in Japan. Omuta City has been learning the lessons of the 2011 Great East Japan Earthquake and Tsunami in Kesennuma City, such as the disaster management and recovery process (Oikawa 2012). Omuta BOE made the best use of appropriate ESD experiences and established links with communities and institutions to make response plans to COVID-19 and to implement them effectively. Under the threat of a spreading COVID-19 infection in Fukuoka Prefecture, Omuta BOE was required to make quick decisions about principles of and action plans for response, direction to schools, and information to parents and citizens. Based on accumulated educational and ESD experience, BOE has implemented continuous responses to prevent COVID-19 from spreading. These plans are adjusted according to the current situation and phase of infection.

#### Phase 1: Japanese government Request for school closure (1 March–6 April 2020)

On 28 February 2020, following the spread of COVID-19 in Japan, the Japanese government requested that all schools in Japan close beginning on 1 March. In a few days, all schools and boards of education in Japan were required to prepare for school closure, to take care of students, and to respond to COVID-19 infections. The Omuta BOE also made some response plans to COVID-19 by communicating and collaborating with school principals and teachers. The BOE held urgent principal meetings many times to discuss preparedness and responses to the pandemic. It conducted planning and implementing responses by utilizing the knowledge and experience in ESD, and it also modified the response of each school while respecting their initiatives.

In response to specific requests at school, teachers visited students’ homes for health checks while schools were closed. The BOE also promoted collaboration with the social welfare section of the city office to support students who needed special assistance, starting a week after school closure. Concretely, Omuta BOE proposed cooperation with the Council of Social Welfare to supply food (lunch box) for underprivileged children during school closure. The BOE and teachers of each school delivered these lunch boxes to students of low-income families. Also the BOE required school principals to invite at-risk students, who might be exposed to child abuse risks in their families, to come to their schools to collect their lunch box in order to check on their lives at home.

In addition, the BOE negotiated with welfare facilities to accept handicapped students while special support schools were closed; the BOE also ordered schools to take care of students of double-income or single-parent families until those children could return to after-school day-care centers, because daytime working parents are unable to provide supervision while children are home. As a further monitoring action, the Omuta BOE dispatched school social workers to low-income households and families with a child abuse history. This initiative created an opportunity to research children’s meal or health situations, and to provide lifestyle support during school closure. Throughout the response, the BOE received advice from specialists, such as a doctor in the public health center, on the best practices available to avoid COVID-19 outbreaks at schools.

#### Phase 2: Declaration of a state of emergency (7 April–14 May 2020)

On 7 April 2020, a state of COVID-19 emergency was declared in Fukuoka Prefecture. Concern about the COVID-19 pandemic was increasing among parents and citizens, because some COVID-19 infected persons were also found in Omuta City. Schools had to shift the method of contact with students from in-person home visits to indirect communication, such as letters or emails, to prevent the spread of the new coronavirus infection. Omuta also directed the curriculum coordinators of each school to reorganize a new school curriculum that could make up for lost learning time because of school closure and adjust to the new school education setting of the post-corona infection period. All the schools reorganized their curriculum and lesson plans by selecting nonacademic school events—such as sports day or culture day—to provide enough lesson hours to learn the reconfigured school curriculum even if the school year was shortened due to school closure.

#### Phase 3: Post declaration of a state of emergency (14–31May 2020)

The emergency declaration in Fukuoka Prefecture was lifted on 14 May 2020. Because COVID-14 was calming down day by day, the BOE could target the day of school restart and schools had to prepare for reopening school. Omuta BOE considered the plan of “distributed school day”^3^ and implemented on 18 May a two week period as a step to “simultaneous school attendance.” On the other hand, on 8 May the BOE decided to shorten summer vacation from 40 to 11 days to recover the delay of learning due to corona infection. Those are very quick responses and decisions. Through a clear decision-making process, which involved consultation with not only teachers but also students and parents, the Omuta BOE was able to build support for an in-person school restart and alleviate concerns about the restructured school education and schedule.

#### Phase 4: School reopening (1 June 2020 onward)

On 1 June 2020, all the schools in Omuta City restarted in-school lessons all at once. In reopening school, each school reconfirmed that ensuring students’ security and safety was the priority of school education. All schools planned to resume school on the premise that corona infection could be prevented. School teachers did their best to build a better environment and lifestyle at each school and classroom to prevent corona infection. For example, practical actions included enforcement of wearing a mask, temperature measurement and gargling, hand washing, disinfection, keeping social distance, improved ventilation, and so on.

Since reopening, new issues have emerged regarding disaster risk reduction at school. In almost all the schools, gymnasiums are designated as evacuation shelters, but they get crowded with evacuees whenever a disaster occurs. Under these conditions, the risk of corona infection inevitably will increase in evacuation shelters from any disaster, so evacuees must maintain social distance even in the shelters. Every municipality, including Omuta City, must find and reserve other evacuation spaces or shelters to supplement school space to prepare for a future disaster, which could occur soon and coincide with a corona virus outbreak. That is a new and common problem for disaster risk reduction to solve during the period of COVID-19.

### Key Learning from Omuta City

The three key issues of pandemic response in the education sector can be reflected through: (1) mitigating the disease impacts on the education sector; (2) preventing increased disease transmission by the education community; and (3) preventing impacts on the education sector from disrupting its educational mission. Table 1 shows key lessons from the Omuta case in these three key pillars of response undertaken by the education sector.

**Table 1:** Key lessons from COVID-19 responses in the education sector of Omuta City

	Mitigating the Impact	Preventing Exacerbation of Transmission	Maintaining Mission Despite Impacts
Phase 1 (Early stage)	Educational governance with school leadership and Board of Education is critical	Specialist advice and science-based decision making is important	Collaboration with other sectors such as social welfare is important
Phase 2 (Emergency stage)	Reorganization of syllabus/curriculum to cover any delay in education delivery is effective	Shifting to online communication instead of direct home visits is found effective	Cancel school events to increase lesson hours enough to enhance learning even in a shortened school year
Phase 3 (Post emergency stage)	Target the day of school restart, and build support of and confidence in Parent and Teacher Association with online discussion	Undertake precaution measures using innovative technologies, like facial temperature check, health check, physical distancing, and so on	Shorten summer vacation to recover lost in-class learning options due to COVID-19 induced school closures
Phase 4 (Recovery stage)	Ensure students security and safety is a priority of school education, including reduced impacts of cascading hazard	Build better environment and lifestyle of school to prevent corona infection	Reserve alternate spaces and shelters to keep social distance for evacuation

A few general lessons emerged from the Omuta City case study can be summarized concisely and also applied in similar conditions when coping with future biological and related cascading hazards:

1. Share information about the COVID-19 pandemic exactly and quickly with the mayor, board members, city council, schools, teachers, students, and parents;
2. Promote principles and policies of the educational response to the prevention of corona virus infection as soon as possible;
3. Clarify the division of roles of BOE, principals, teachers, and parents;
4. Visualize the decision-making process to field based responses;
5. Introduce the knowledge and advice of epidemiology specialists into decision-making and establish rigorous response processes to maximize prevention of corona infection; and
6. Create a proactive planning and response process that is mutually supportive of and compatible with disaster risk reduction management

## Discussion

The new realization of DRR education lies in the response to new types of hazard such as biological hazards. Although the Sendai Framework has expanded the scope of hazards to biological hazards, NaTech (natural hazard induced technological disaster), and so on, this is the first time in recent history that we have faced such a prolonged biological hazard in terms of pandemic, which has affected different parts of global society. Cascading disasters like typhoons, floods, and heat waves also have been prominent during the global pandemic in Japan as well as elsewhere. In a recent report, Das et al. (2020) discussed evacuation and shelter management challenges during floods in west Japan amidst the pandemic. Usually the schools become evacuation shelters during disasters in Japan, which created critical challenges of shelter management during the COVID-19 outbreak. In 2020, people did not evacuate to schools in many cases, mainly due to fear of infection and a desire not to affect the education continuity (because most of school classes are already affected due to pandemic). Where people have evacuated into the schools—mostly abandoned school buildings in rural and semirural areas in Kumamoto Prefecture—shelter management was a key challenge due to the new government guideline of 60% occupancy of the shelter to maintain safe physical distancing. Also, a strict health monitoring mechanism was imposed, and the entry of volunteers was restricted. The lessons from cascading disasters, as compiled by Das et al. (2020), will be useful for future school operation during time of emergency. New learning of DRR education can be summarized below in terms of (1) governance and decision making; (2) school-community-family linkages; (3) risk communication and citizen behavior; and (4) use of technology.

Educational governance becomes very critical during as well as after pandemic decision making, where crucial decisions on the opening of schools and maintenance of appropriate social behavior inside and outside school (during commuting) become important. All three aspects of educational governance (structural, nonstructural, and functional) issues become crucial. In many countries COVID-19 related education is primarily categorized under health and sanitation education for washing hands taking proper care of health issues, and so on. But a proper integration into DRR education is desirable. Also, linkage of the Board of Education with the Social Welfare department helped in addressing the issues of the vulnerable children.

The connection between school and community, including families, is very critical for promoting DRR education. As a lesson of the 2011 Great East Japan Earthquake and Tsunami, it was reported that disaster management, including evacuation and management of shelter, was remarkably successful in those schools that have good linkages and collaborations with their communities and outside institutions through high quality DRR education within the community (Oikawa 2013). From the case of Omuta City, significant commonalities with school responses and education to prevent disease spread during the COVID-19 pandemic can be observed. The school is not able to prevent the spread of corona infection without cooperation with community and family, because the school had to close for a while and to entrust students to their families and communities as the COVID-19 virus rapidly diffused globally and locally. In Japan, the linkage of school and community must be built through collaborative school activities or joint projects with community. The Parent and Teacher Association (PTA), working in a collaborative system with the local community, was able to achieve broad support for a Community School. Education for sustainable development embraces all of these initiatives and it also contributes to create a sustainable society and community; this is the best approach and method to build linkages and community engagement (Oikawa 2014a). Thus, the key essence of ESD and DRR education as school community linkage is reestablished through the lessons from pandemic responses, and this leads to achievement of sustainable development goals and emergence of a more resilient society and community.

Risk communication becomes an important factor, and schools can play a vital role by enhancing risk communication (Shaw et al. 2014). For DRR education, providing appropriate information through schools to community and family is effective. In case of pandemic response also, it was found an effective mechanism to provide the right and trusted information. A pandemic is an invisible disaster, correct information from a trusted source is crucial, and schools have played important roles in information dissemination. Behavioral changes and being responsible citizens are of utmost importance, which is linked to school and community collective education (Shaw, Kim, et al. 2020).

Finally, COVID-19 has seen the use of different types of innovative technologies in all sectors, including education. Several innovative ways to provide online classes and online classrooms were used to deliver lectures and maintain teacher and student contact. Health surveillance using a thermal camera was common in most of the schools. Different apps were used extensively for contact tracing in many schools and education sectors. All these technologies have implications for DRR education as well, which can also be used for education continuity through online classes (when schools are used as evacuation shelter after a disaster) or safety notification after a disaster through health related apps, as well as regular health checks, and so on.

What could be the implication of the COVID-19 experience to disaster education? There is an urgent need for a conversion about disaster prevention education that promotes a true disaster risk reduction and resilience education in the new risk landscape. Focusing on the Sendai Framework priority, understanding that risk includes all its dimensions of vulnerability, capacity, exposure of persons and assets, hazard characteristics, and the environment. This holistic approach needs to be included in risk reduction and resilience education (UNDRR 2017a). The current concept of single hazard DRR education also needs to be changed. We are increasingly facing cascading hazards and complex emergencies with different levels of uncertainties. Thus, traditional DRR education is significantly challenged and we need new cycles of learning for holistic DRR and resilience education. Risk communication for complex emergencies, capacity enhancement, and resilience building are some important key issues.

## Conclusion

The Sendai Framework has expanded the scope of hazards, and thus DRR education needs to incorporate new learning from COVID-19 responses in the education sector. Cascading risks also become a crucial factor in the prolonged timeframe of a global pandemic. COVID-19 has seriously affected the education sector, and its responses are also varied based on each country’s governance structure. The case of Japan presented here has drawn the lessons from the evolution of DRR education, previous learning from the 2011 East Japan Earthquake and Tsunami, and the current pandemic. The experience of Omuta City, which is a champion city of ESD, shows that SDGs are attainable as a part of a holistic strategy and that governance plays a critical role in education in emergencies. Informed and science-based decision making by the Board of Education and schools are critical both in the pandemic as well as in DRR education. School-community-family linkages, risk communication, and responsible citizen behavior are key variables to reduce the impacts of the pandemic disaster and can be adapted to other disaster risk management needs. Technology has played a critical role in the COVID-19 pandemic, which can also be used in future DRR education. New realization of DRR education lies in the fact that it offers an essential lens through which to understand a complex emergency with cascading risks and differential levels of uncertainties. Also, health education and DRR education have complementarities, but still need to promote more synergies. Decision making and risk communication for complex emergencies needs to be incorporated in DRR education, so that it becomes a comprehensive risk reduction and resilience education, leading to a safer and sustainable society and community.

## Footnotes

1 https://en.unesco.org/news/esd-2030-whats-next-education-sustainable-development.

2 https://www.mext.go.jp/unesco/004/1339970.htm.

3 This means that students are grouped in different sections, and were asked to come to school alternate days to avoid the concentration of people in one place.
